# Supplementary material for: Mechano-adaptive meta-gels through synergistic chemical and physical information-processing
Source: Nat Commun. 2024 Oct 17;15:8957. doi: 10.1038/s41467-024-53368-1 (PMC11487081; doi:10.1038/s41467-024-53368-1)
Supplement: Supplementary file 1 — Supplementary Information [file 41467_2024_53368_MOESM1_ESM.pdf]

Supplementary Information

Mechano-Adaptive Meta-Gels Through Synergistic Chemical and Physical Information-Processing

Brigitta Dúzs, Oliver Skarsetz, Giorgio Fusi, Claudius Lupfer and Andreas Walther**

Life-Like Materials and Systems, University of Mainz, Duesbergweg 10-14, Mainz, 55128, Germany

E-mail: brigitta.duzs@uni-mainz.de; andreas.walther@uni-mainz.de

Contents

Supplementary Notes 2

1. Urea-urease front layer 2
2. pH-sensitive response layer 2
3. Initiator patch 2

Supplementary Figures 3

Supplementary Notes

1. Urea-urease front layer

The urease-containing layer was made of sPEG, where the gelation happens in a click reaction. In any free-radical photopolymerized pAAm-based gel the urease enzyme would suffer a loss of activity because of a reaction with the radicals.

2. pH-sensitive response layer

The pH-sensitive response layer was made with PEGDA6k crosslinker to achieve similar water affinity in the two sides of the bilayer in the initial state and avoid initial bending that is not related to the front but is only the consequence of the water-equilibration between the materials. Theoretically, the sPEG is an ideal network hydrogel, so it has a large swelling capacity. The PEGDA6k is a bivalent, large molecular weight crosslinker, so the amount of solubilized water relative to the moles of crosslinks is higher than with Bis. Consequently, the use of PEGDA6k in the pAAm response layer allows for higher water uptake without the dramatic decrease in gel consistency, which is important for device fabrication and handling.

3. Initiator patch

The high-pH patch was made from DMAEAAm hydrogels instead of loading with NaOH to avoid activation by diffusion via the connecting neck of the activation patch. The pDMAEAAm is a base, in aqueous solution it forms OH^- and a polycation. The polycation has limited diffusivity due to its size and thus it minimizes the diffusion of the counterion OH^- due to electrostatic interaction. In Fig. 4, additional isolation made of p(AAm-*co*-AA-*co*-PEGDA6k) containing 100 mM AA was used in the neck above the DMAEAAm patch to protect the body of the strain gate from parasitic activation. In Fig. 4b at 120 min, the small activation patch is already depleted (yellow) by the buffering of the low pH isolating neck, but the front keeps propagating, confirming the self-sustaining nature of the RD front.

Supplementary Figures

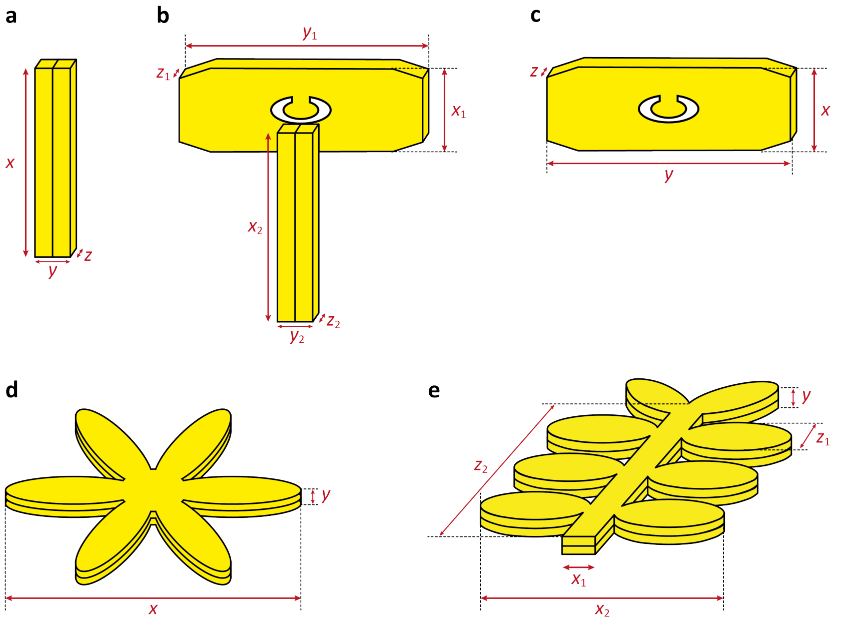

Figure S1. Dimensions of the fabricated hydrogel objects. (a) Bilayer actuator: $x = 20$ mm, $y = 1.5$ mm, $z = 2.0$ mm. (b) Strain gate combined with bilayer actuator: $x_1 = 9$ mm, $y_1 = 27$ mm, $z_1 = 1.0$ mm, and $x_2 = 25$ mm, $y_2 = 1.5$ mm, $z_2 = 2.0$ mm. (c) Strain gate for gel strengthening: $x = 9$ mm, $y = 27$ mm, $z = 1.0$ mm. (d) Bilayer gripper: $x = 14$ mm, $y = 1.5$ mm. (e) Mimosa: $x_1 = 2$ mm, $x_2 = 15$ mm, $z_1 = 2.5$ mm, $z_2 = 15$ mm, and $y = 1.5$ mm.

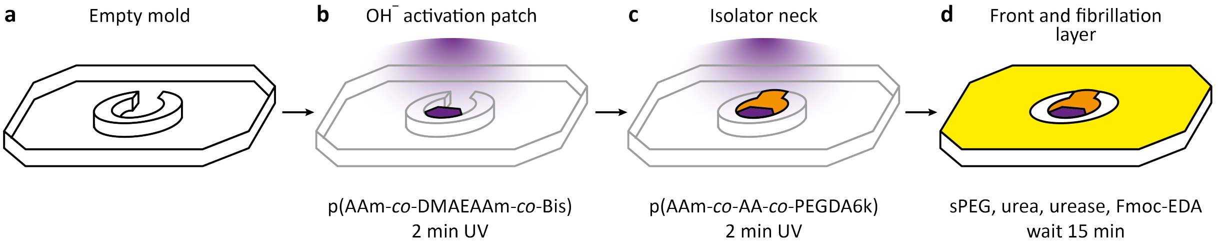

Figure S2. Example of multi-material hydrogel device preparation with sequential molding of different resin mixtures providing different functionalities in the final device. (a) The empty Teflon mold made with CNC machine is the inverse of the wanted final gel structure. (b) The first resin solution (containing monomers, crosslinker, LAP, and indicator) is pipetted in the corresponding mold part and solidified under UV lamp in 2 min. (c) If applicable, the next photopolymerizable resin solution is pipetted in the corresponding mold part and placed under the UV lamp quickly. (d) The solution of sPEG macromonomers and components of the reaction network is pipetted in the rest of the mold and gelates by waiting for 15 min. The resulting multimaterial piece is gently removed from the mold and placed into silicone oil for the experiments.

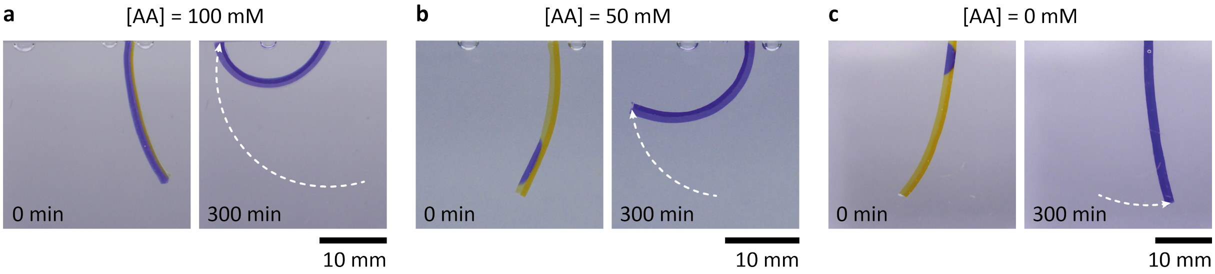

Figure S3. Actuation efficiency in the bilayer depends on the acrylic acid (AA) concentration of the response layer. Snapshots right after the device preparation (0 min) and after completed actuation (300 min) in case of (a) 100 mM AA, (b) 50 mM AA, and (c) 0 mM AA. The higher the concentration of the pH-responsive AA monomer, the larger the bending. We used the same chemical composition in the front layer in all cases, but with relatively high urease concentration to ensure fast (self-initiated) OH^- accumulation. The response layer was made of 1100 mM AAm and 11 mM Bis instead of PEGDA6k crosslinker to avoid hydrolysis at the applied high pH.

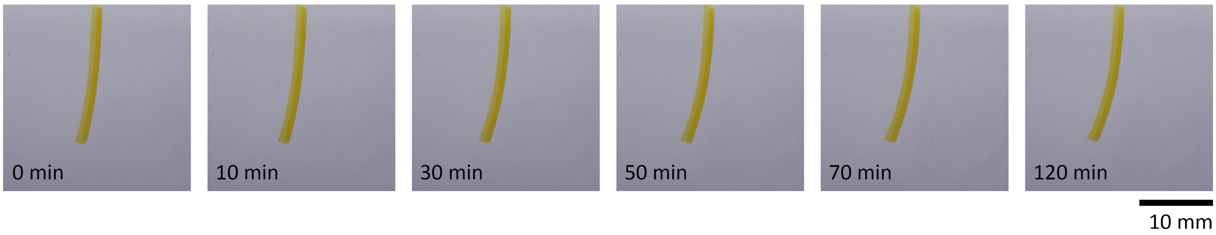

Figure S4. Control experiment for bilayer actuators without OH^- front propagation. The front layer contains no urease while the response layer has the same pH-responsive composition as in Fig. 2i. In the absence of basification, no bending was observed.

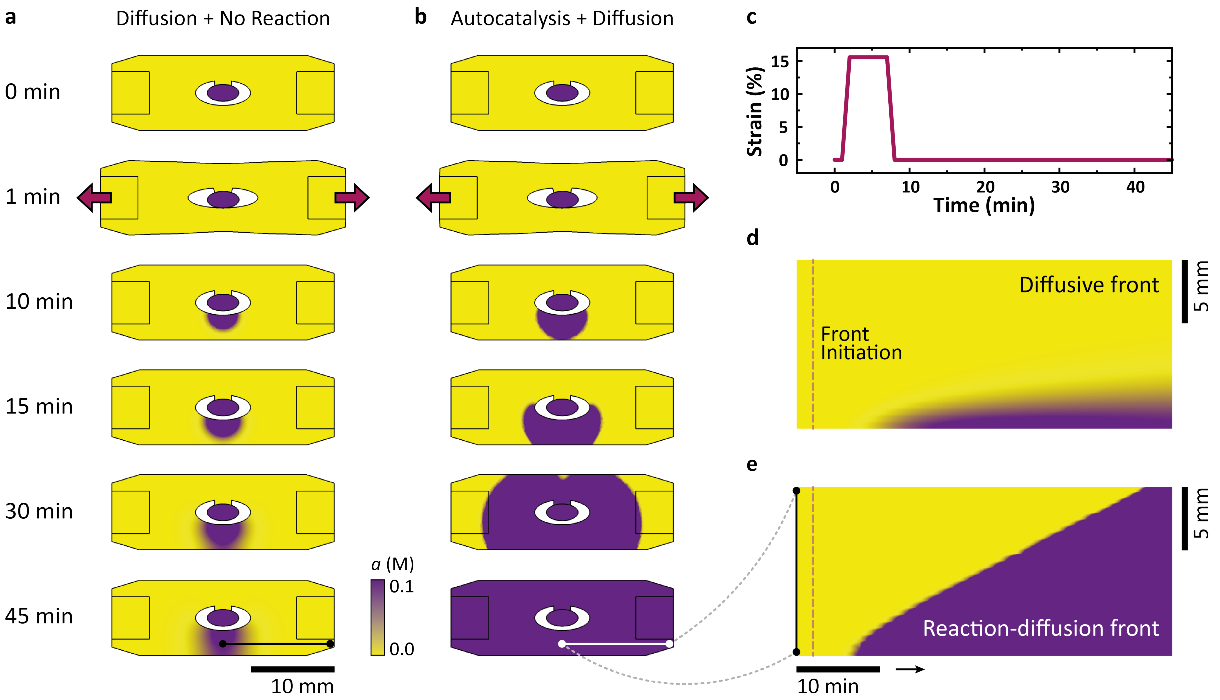

Figure S5. Finite element simulations of the strain gate and chemical transport mechanisms. (a) Simple diffusive front propagation, (b) RD front due to an autocatalytic process. (c) Temporal strain profile during stretching. Propagation efficiency of (d) diffusive and (e) RD signals in kymographs made along the line segments in panels (a-b). The time of the front initiation at the touching point is indicated by dotted lines.

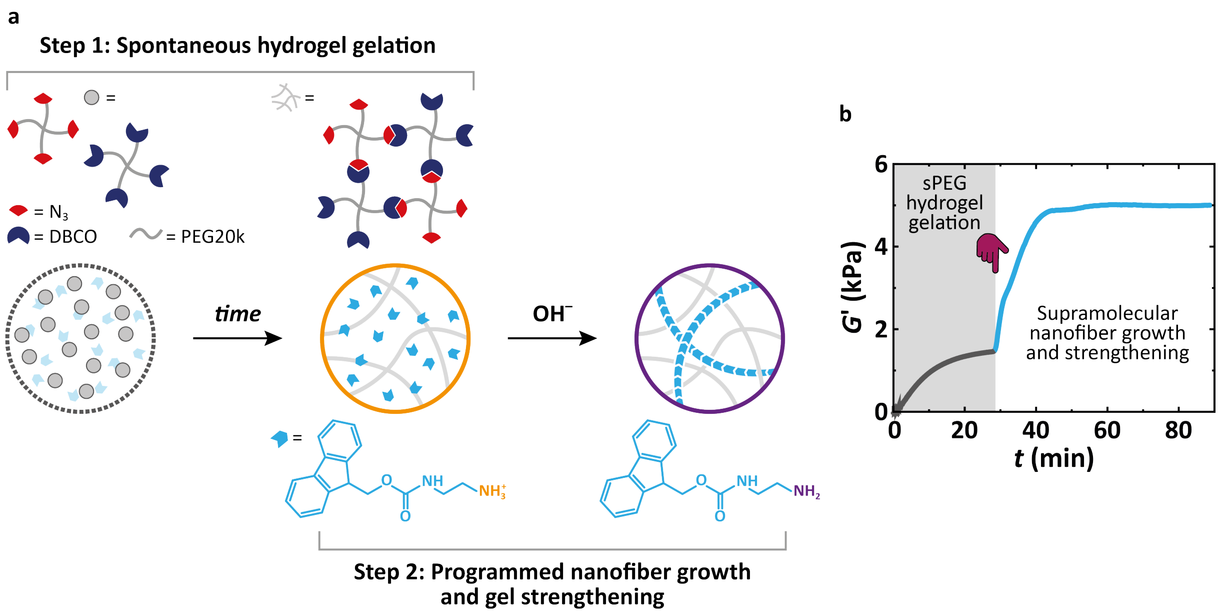

Figure S6. Structural changes in the hydrogel during the entire process from device fabrication to front-induced strengthening. (a) Chemical reactions and scheme of macromolecular changes. Step 1: Spontaneous sPEG gelation and formation of the basis material of the gel device. The pregel liquid mixture contains macromonomers (grey circles) that form a crosslinked polymer network (grey lines). Step 2: When we externally initiate the urea-urease front with a small droplet of 0.1 M NaOH, the autocatalytically produced OH^- deprotonates the Fmoc-EDA (blue). The self-assembly of the deprotonated molecules forms an additional fiber network. (b) Temporal evolution of the storage modulus (*G*') measured in oscillatory shear rheology starting from the sPEG pregel mixture. The red hand indicates the front initiation. While the OH^- front propagates through the sample in 2 min (detected visually), the *G*' keeps changing for 10-15 min until it eventually plateaus 3.3 times above the original *G*'.
