# Supplementary material for: Mechano-adaptive meta-gels through synergistic chemical and physical information-processing
Source: Nat Commun. 2024 Oct 17;15:8957. doi: 10.1038/s41467-024-53368-1 (PMC11487081; doi:10.1038/s41467-024-53368-1)
Supplement: Supplementary file 2 — Description of Additional Supplementary Files [file 41467_2024_53368_MOESM2_ESM.pdf]

Description of Additional Supplementary Files

Supplementary Movie 1

Touch-Activated Autonomous Bilayer Actuator

Local mechanical trigger (touch) is transformed to a chemical signal, which propagates as a reaction-diffusion front. Eventually it leads to global structural adaptation via polyelectrolyte gel swelling, and the bilayer actuator bends.

Supplementary Movie 2

Mechano-Adaptive Soft Robots

We present touch-sensing and signal-processing bilayer actuators with complex 2D shapes. In these devices, a hydrogel domain with a reaction-diffusion signaling front and a pH-responsive hydrogel domain are combined for biomimetic self-protective behavior.

Supplementary Movie 3

Complex Multi-Material Gel Device with Mimosa-Like Mechano-Adaptation

These soft robots imitate the mechanically triggered self-protective movements seen in plants like the mimosa. Thanks to the multi-material design, the stem remains straight, but the perpendicular pairs of leaves bend and close as the front propagates.

Supplementary Movie 4

Metamaterial Strain Gate Design by Finite Element Simulations

Tensile force is applied horizontally on the gel object. At lower strains, the activator patch does not touch the bottom main body, there is no mechano-activation. Above the strain threshold, a local touching happens and initiates global signal transmission, even after the release of the strain.
